# Tri-ponderal mass index and left ventricular hypertrophy in a cohort of caucasian children and adolescents with obesity

**DOI:** 10.1186/s13052-024-01634-9

**Published:** 2024-04-19

**Authors:** Sara Vizzuso, Alberico Del Torto, Giulia Fiore, Stefano Carugo, Gianvincenzo Zuccotti, Elvira Verduci

**Affiliations:** 1 Department of Pediatrics, Buzzi Children’s Hospital, University of Milan, Milan, Italy; 2 Centro Cardiologico Monzino IRCCS, Milan, Italy; 3 Department of Health Sciences, University of Milan, Milan, Italy; 4 Department of Internal Medicine, Cardiology Unity, University of Milan, Fondazione Ospedale Maggiore IRCCS Policlinico Milano, Milano, Italy; 5 Department of Clinical Sciences and Community Health, University of Milan, Milan, Italy; 6 Department of Biomedical and Clinical Sciences, University of Milan, Milan, Italy

**Keywords:** Pediatric obesity, Cardiovascular risk, Tri-ponderal mass index, Left ventricular hypertrophy, Left ventricular mass index

## Abstract

**Background:**

Pediatric obesity is a global emerging burden for society; among its health-related consequences there are hypertension (HTN) and left ventricular hypertrophy (LVH). Several anthropometric indices have been investigated for the early identification of cardiovascular risk in children. The aim of the present study was to assess whether tri-ponderal mass index (TMI) was associated with LVH in a cohort of Caucasian children and adolescents with obesity.

**Methods:**

In this observational study, 63 children and adolescents with obesity aged 7-to-16 years were enrolled. During outpatient visits, adiposity, and cardio-metabolic indices (BMI z-score, WHR, TMI, ABSI) were collected. All subjects underwent a 24-hour ambulatory blood pressure monitoring (ABPM) and transthoracic echocardiography.

**Results:**

Children and adolescents with obesity with LVH had significantly higher BMI z-score (*p* = 0.009), WHR (*p* = 0.006) and TMI (*p* = 0.026) compared to children without LVH. WC and WHR were the only indices significantly associated with left ventricular mass index (LVMI).

**Conclusion:**

Left ventricular remodeling is associated with the cardio-metabolic risk markers WC and WHR, but not with the adiposity index TMI among children with obesity.

**Supplementary Information:**

The online version contains supplementary material available at 10.1186/s13052-024-01634-9.

## Introduction

Obesity is the most prevalent chronic disease among children worldwide and it is a global significant burden for society [1]. Obesity can have many consequences on health, particularly Metabolic Syndrome (MetS) [2], described as a cluster of cardiovascular (CVD) risk factors such as abdominal obesity, hyperglycaemia, dyslipidaemia and hypertension (HTN) [3]. The known association between childhood BMI and adult cardio-metabolic diseases may reflect a common genetic predisposition, but could also be related to BMI persistence from childhood to adulthood [4].

HTN in children with obesity is a strong predictor of HTN in adulthood [5], and when it occurs during childhood or adolescence, it usually persists in adulthood even if Body Mass Index (BMI) is normalized into normal ranges [6]. To identify children at risk, along with office blood pressure (BP) measurement, 24-hour ambulatory BP monitoring (ABPM) is a useful diagnostic tool.

Observational studies confirm a greater left ventricular mass, left ventricular septal and posterior wall thickness, and left ventricular end-diastolic diameter in children with obesity when compared to lean subjects, even in subjects without hypertension [7–9]. The association of obesity and abnormal left ventricular geometry and function is debated; it is not clear whether it is the obesity itself to determine left ventricular alterations, or whether there is an intermediate player such as HTN or other subtle vascular changes [10].

Different anthropometric indices that are surrogates of body composition have been investigated for the early identification of CVD risks. BMI cannot accurately depict body composition in the pediatric population, as weight and height squared are not proportional during some growth phases [11]. Also BMI z-score, known as the standard indicator for screening paediatric obesity, showed to be only weakly associated with other measures of body fatness in paediatric age [12].

Therefore Peterson et al. proposed the Tri-ponderal Mass Index (TMI) as a replacement for BMI z-score in screening for body fat in children and adolescents [13]. Interestingly, TMI demonstrated a lower rate of misclassification than BMI in detecting body fat and HTN in overweight adolescents [13]. Notably TMI was proposed as a more reliable index among overweight and children with obesity [13–15]. Additionally, in our previous work we found TMI as being a good predictor of high blood pressure among males older than 10 years of age [16], although we assessed exclusively office blood pressure in an outpatient setting.

A Body Shape Index (ABSI) has been showed to have considerable associations with cardiometabolic risk markers in children and adolescents with obesity [16–19].

Up to date no studies have investigated the association between TMI or ABSI and left ventricular hypertrophy in pediatric age groups.

Conversely, evidence is emerging regarding the potential implication of the Waist-to-height ratio (WHR). WHR predicts abdominal fat mass and takes body size into account [20] and a value ≥ 0.55 is a proper cut-off for screening European children at high cardiometabolic risk [21, 22]. WHR has certain advantages over WC alone, since having a universal cut-off would help standardize practice, which may be independent of age, sex, height and race [21]. Moreover its utility in discriminating children with cardiac structural damage, mainly left ventricular hypertrophy (LVH) and left ventricular geometry (LVG), has been reported [23, 24].

The aim of this observational study was to assess whether TMI was associated with LVH in Caucasian children and adolescents affected by obesity. Secondarily, we sought to identify anthropometric, adiposity and glycometabolic indices that predicted left ventricular mass index (LVMI) in a population accurately screened using ABPM and echocardiography.

## Materials and methods

### Subjects and design

This was an observational study conducted from September 2016 to February 2018 based on a cohort of consecutive Caucasian children and adolescents with obesity enrolled at the outpatient settings of the Paediatric Department of the San Paolo Hospital in Milan (Italy) as already described [9]. To be eligible to participate in the present study, individuals had to respect the following inclusion criteria:

1. between 7-to-16 years of age;
2. diagnosis of obesity according to World Health Organization criteria (BMI z scores for age and gender ≥ + 2 Standard Deviations) [25];
3. agreement by the individual and their legal guardian to fully participate in the assessments;
4. absence of secondary obesity, familiar dyslipidaemia with glucose metabolism alterations, diabetes mellitus, hypertension, pre-existing cardiovascular diseases and genetic or endocrine dysfunction.

After enrollment, all participants underwent a complete examination in the pediatric obesity outpatient clinic, including a medical visit, anthropometric assessments, repeated office BP measurements, echocardiogram, and 24-hour ABPM.

This study was approved by the hospitals’ Institutional Review Boards and all procedures were conducted in line with the Declaration of Helsinki. The study was conducted in accordance with the local medical ethical committee (protocol number 2015/ST/135).

At recruitment, all participants and their legal guardian provided written informed consent.

### Anthropometric assessments and adiposity indices

After enrollment all children underwent physical examination, as previously described [9]. Anthropometric assessment, including body weight, height and waist circumference (WC) measurements, were performed by well-trained health professionals. BMI was calculated as:

$${\text{BMI = Weight(kg)/[Height(m)}}{{\text{]}}^{\text{2}}}$$

BMI values were standardized into BMI z-scores, using WHO reference values [25].

To better assess cardio-metabolic risk, WHR index was calculated as [26]:

$${\text{WHR = WC}}\,{\text{(m)/Height(m)}}$$

TMI was calculated according to the following formula:

$${\text{TMI = Weight(kg)/[Height(m)}}{{\text{]}}^{\text{3}}}$$

ABSI was calculated as [27]:

$${\text{ABSI}}\,=\,{\text{WC(m)}}/[{{\text{BMI}}\,({\text{Kg}}/{{\text{m}}^{2}})]^{2/3}}\, \times \,{{\text{[Height(m)]}}^{1/2}}$$

### Biochemical parameters and glycometabolic indices

Insulin and fasting blood glucose were determined by venous blood sampling after twelve hours of fasting. The glycometabolic indices Homeostatic Model Assessment Index– Insulin Resistance (HOMA-IR), HOMA of β-cell function (HOMA-β) and QUantitative Insulin-sensitivity Check Index (QUICKI) were calculated. These glycometabolic indices are used in clinical practice to detect children and adolescents at risk of type 2 diabetes mellitus calculated as previously described [9].

### Blood pressure and 24-hour ABPM

Detailed description of procedure are described elsewhere [9]. In brief, office BP was measured by means of mercury sphygmomanometer and stethoscope according to European guidelines [28]. Systolic BP (SBP) and diastolic BP (DBP) values were the average of the last two mesaurments. The percentiles of SBP and DBP were calculated by reference normograms distributed by sex, age and height in accordance with the recommendations provided by the National High Blood Pressure Education Program Working Group on High Blood Pressure in Children and Adolescents and the European Society of Hypertension [28, 29].

The 24 h ABPM exam was collected by means of Custo screen 300 med (Sylcomed, Sylco S.r.l.) device. 24-hour, daytime and night-time mean SBP and DBP were recorded and evaluated according to American Heart Association (AHA) recommendations [30]. According to 24 h-ABPM results children were classified as normotensive, if mean 24-h BP values were normal, or hypertensive.

### Echocardiography

Subjects affected by obesity underwent transthoracic echocardiography, as already described [9]. The exams were performed by the same operator. Transthoracic echocardiography was conducted to obtain left ventricular end-diastolic diameter, interventricular septum and posterior wall thickness, by averaging standard M-mode measurements in three different cardiac cycles according to the American Society of Echocardiography recommendations [31]. Left ventricular mass was estimated using the Devereux formula [32]. Left ventricular mass index (LVMI, g/m2) and relative wall thickness (RWT) were calculated according to the American Society of Echocardiography indications [31]. Age- and sex-corrected LVMI percentiles were used [33] and age-corrected RWT values above 0,390 were considered abnormal [34]. Left ventricular hypertrophy was defined as LVMI or RWT equals or greater than 95th percentile for age and sex [28].

### Statistical analysis

Continuous variables were expressed as median (interquartile range) as they were not normally distributed according to normality tests. Discrete variables were reported as frequency and percentage. Mann-Whitney U test and χ2 test were used as appropriate to compare characteristics of patients affected by obesity with and without left ventricular hypertrophy and of hypertensive and non-hypertensive patients with obesity (according to ABPM readings). Spearman correlation test was used to assess continuous variables correlations. Multiple testing adjustment was performed according to Bonferroni-Holm. Sex- and age-adjusted logistic multivariable analysis models were used to assess the association between BMI z-score, TMI, WC, WHR or ABSI with presence of left ventricular hypertrophy. McFadden pseudo-R2 was used as a measure of association. P values < 0.050 were considered statistically significant. Statistical analyses were performed using SPSS Statistics version 20 (IBM Corp., Armonk, NY).

## Results

Table 1 shows anthropometric, adiposity and glycometabolic indices, office SBP and DBP, main echocardiography and ABPM parameters of patients affected by obesity. Sixty-three children and adolescents with obesity were enrolled. The median age of the patients was 11 years. Fourteen patients were diagnosed with LVH at echocardiography and 35% of patients were diagnosed as hypertensive at ABPM. Table 1 also illustrates gender differences. Twenty-one (49.2%) patients were females. The BMI z-score was not significantly different between the two groups. Twenty-five (78%) males and twenty-four females (77%) with obesity were diagnosed with LVH at echocardiography while 56% of males and 74% of females were diagnosed as hypertensive at ABPM.

Patients were grouped according to presence of LVH at echocardiography. Table 2 compares the above-mentioned indices in patients with and without LVH. Children and adolescents with obesity with LVH had significantly higher BMI z-score, TMI and WHR. HOMA-β was significantly higher in patients with LVH. Mean 24-hour SBP and DBP did not differ significantly between patients with and without LVH (Table 2).

**Table 1:** Characteristics of the population and of male and female patients

	Cohort		Females		Males		P value
	(*n* = 63)		(*n* = 31)		(*n* = 32)		
Age (years)	11	(10–12)	11	(10–12)	11	(9–12)	0.961
Females	31	(49.2%)					
BMI z-score	2.64	(2.46–3.04)	2.65	(2.25–3.04)	2.62	(2.47–3.05)	0.437
TMI (Kg/m^3^)	18.01	(16.87–19.68)	18.51	(17.09-19-92)	17.64	(16.48–19.26)	0.093
WC (cm)	88	(83–96)	87	(83–96)	89	(82–96)	0.544
WHR	0.59	(0.56–0.62)	0.59	(0.55–0.63)	0.59	(0.57–0.62)	0.564
ABSI	0.080	(0.076–0.082)	0.077	(0.074–0.081)	0.081	(0.078–0.083)	0.006*
ABSI z-score	0.223	(-0.697, 0.837)	-0.449	(-1.209, 0.500)	0.629	(-0.113, 1.173)	0.005*
HOMA-IR	3.34	(2.22–5.06)	3.69	(2.26–5.98)	3.34	(2.17–4.51)	0.326
HOMA β	260.1	(194.1-333.6)	293.6	(216.0-447.7)	251.2	(142.0-285.6)	0.025*
QUICKI	0.32	(0.30–0.34)	0.31	(0.30–0.34)	0.32	(0.31–0.34)	0.256
Office SBP (mmHg)	114	(117–120)	113	(110–120)	115	(107–123)	0.705
Office DBP (mmHg)	62	(58–60)	60	(58–70)	64	(59–70)	0.388
IVSd (cm)	0.7	(0.7–0.8)	0.7	(0.7–0.8)	0.7	(0.7–0.8)	0.762
LVEDD (cm)	4.2	(4.0-4.5)	4.2	(3.7–4.3)	4.4	(4.0-4.6)	0.012*
LVPWd (cm)	0.7	(0.7–0.8)	0.7	(0.6–0.8)	0.7	(0.7–0.8)	0.248
EF (%)	64	(62–65)	64	(62–66)	63	(61–65)	0.229
LVMI (g/m^3^)	29.8	(26.0–33.4)	27.5	(24.9–32.4)	30.8	(27.9–35.6)	0.012*
LV Hypertrophy	14	(22.2%)	24	(77%)	25	(78%)	0.592
Mean 24-hour SBP (mmHg)	111	(106–117)	110	(105–116)	112	(106–119)	0.417
Mean 24-hour DBP (mmHg)	70	(67–73)	69	(66–72)	71	(69–74)	0.035*
Mean daytime SBP (mmHg)	117	(113–123)	115	(111–121)	119	(114–125)	0.090
Mean daytime DBP (mmHg)	75	(72–80)	74	(70–78)	77	(72–82)	0.014*
Mean night-time SBP (mmHg)	101	(96–109)	100	(97–108)	103	(95–110)	0.934
Mean night-time DBP (mmHg)	61	(59–65)	61	(59–63)	63	(58–67)	0.151
Hypertension at ABPM	22	(35%)	23	(74%)	18	(56%)	0.109
Non dipper	18	(28.6%)	20	(65%)	25	(78%)	0.180

Body Mass Index z-score (BMI z-score), Tri-ponderal Mass Index (TMI), Waist Circumference (WC), Waist-to-Height Ratio (WHR), A Body Shape Index (ABSI), Homeostatic Model Assessment Index– Insulin Resistance (HOMA-IR), Homeostatic Model Assessment Index -β (HOMA-β), Quantitative Insulin sensitivity Check Index (QUICKI), Systolic Blood Pressure (SBP), Diastolic Blood Pressure (DBP), Interventricular Septum thickness (IVSd), Left Ventricular Internal Dimensions at end Diastole (LVEDD), Left Ventricular Posterior Wall thickness (LVPWd), Ejection Fraction (EF), Left Ventricular Mass Index (LVMI), Left Ventricular (LV), Ambulatory Blood Pressure Monitoring (ABPM), * *P* < 0.050

Anthropometric, adiposity and glycometabolic indices, office SBP and DBP, main echocardiography and ABPM parameters were compared in patients with and without hypertension at ABPM (Table 3). WC was significantly higher in patients with hypertension, as HOMA-IR and HOMA-β, while QUICKI was significantly lower in hypertensive patients. LVMI and prevalence of LVH did not differ significantly between hypertensive and non-hypertensive patients (Table 3).

**Table 2:** Comparison between patients affected by obesity with and without left ventricular hypertrophy

	LVH		No LVH		P value
	(*n* = 14)		(*n* = 49)		
Age (years)	10	(9–12)	11	(10–12)	0.240
Females	7	(50%)	24	(49%)	1.000
BMI z-score	3.12	2.56–3.53)	2.61	(2.44–2.96)	0.009*
TMI (Kg/m^3^)	19.02	(17.36–21.73)	17.80	(16.59–19.37)	0.026*
WC (cm)	93	(83–98)	88	(81–96)	0.192
WHR	0.63	(0.59–0.65)	0.59	(0.55–0.61)	0.006*
ABSI	0.080	(0.077–0.082)	0.080	(0.075–0.082)	0.698
ABSI z-score	0.232	(-0.600, 0.849)	0.223	(-0.872-0.960)	0.840
HOMA-IR	4.30	(2.65–6.37)	3.24	(2.18–4.90)	0.074
HOMA β	315.7	(252.6-450.7)	247.8	(173.1-316.7)	0.016*
QUICKI	0.31	(0.29–0.33)	0.32	(0.30–0.34)	0.085
Office SBP (mmHg)	120	(111–125)	111	(106–120)	0.061
Office DBP (mmHg)	62	(60–74)	61	(57–70)	0.450
IVSd (cm)	0.8	(0.7–0.9)	0.7	(0.7–0.8)	0.014*
LVEDD (cm)	4.1	(3.8–4.3)	4.3	(4.1–4.5)	0.040*
LVPWd (cm)	0.8	(0.7–0.9)	0.7	(0.6–0.8)	0.003*
EF (%)	62	(60–64)	64	(62–66)	0.055
LVMI (g/m^3^)	33.7	(26.2–38.5)	28.4	(26.0-32.5)	0.032
Mean 24-hour SBP (mmHg)	116	(106–122)	110	(106–116)	0.100
Mean 24-hour DBP (mmHg)	71	(68–75)	70	(67–73)	0.461
Mean daytime SBP (mmHg)	122	(114–127)	115	(113–122)	0.050
Mean daytime DBP (mmHg)	76	(71–83)	75	(72–79)	0.393
Mean night-time SBP (mmHg)	108	(100–113)	100	(96–108)	0.056
Mean night-time DBP (mmHg)	63	(57–65)	61	(59–64)	0.636
Hypertension at ABPM	6	(42.9%)	16	(32.7%)	0.534
Non dipper	4	(28.6%)	14	(28.6%)	1.000

Body Mass Index z-score (BMI z-score), Tri-ponderal Mass Index (TMI), Waist Circumference (WC), Waist-to-Height Ratio (WHR), A Body Shape Index (ABSI), Homeostatic Model Assessment Index– Insulin Resistance (HOMA-IR), Homeostatic Model Assessment Index -β (HOMA-β), Quantitative Insulin sensitivity Check Index (QUICKI), Systolic Blood Pressure (SBP), Diastolic Blood Pressure (DBP), Interventricular Septum thickness (IVSd), Left Ventricular Internal Dimensions at end Diastole (LVEDD), Left Ventricular Posterior Wall thickness (LVPWd), Ejection Fraction (EF), Left Ventricular Mass Index (LVMI), Left Ventricular (LV), * *P* < 0.050

**Table 3:** Comparison between patients affected by obesity with and without hypertension according to ABPM

	Hypertensive		Non-hypertensive		P value
	(*n* = 22)		(*n* = 41)		
Age (years)	12	(10–12)	10	(10–12)	0.083
Females	8	(36.4%)	23	(56.1%)	0.188
BMI z-score	2.85	(2.47–3.07)	2.61	(2.45–3.01)	0.360
TMI (Kg/m^3^)	17.91	(16.98–19.20)	18.01	(16.69–19.80)	0.994
WC (cm)	94	(86–98)	86	(80–94)	0.013*
WHR	0.59	(0.56–0.62)	0.59	(0.56–0.62)	0.659
ABSI	0.080	(0.078–0.081)	0.079	(0.074–0.084)	0.819
ABSI z-score	0.274	(-0.327-0.694)	0.168	(-1.121-1.265)	0.680
HOMA-IR	4.21	(3.34–5.95)	2.65	(2.14–4.75)	0.018*
HOMA β	283.2	(244.8-362.6)	247.8	(157.1-324.6)	0.037*
QUICKI	0.31	(0.30–0.32)	0.33	(0.31–0.34)	0.018*
Office SBP (mmHg)	120	(114–126)	111	(105–117)	0.002*
Office DBP (mmHg)	62	(60–67)	60	(57–70)	0.783
IVSd (cm)	0.80	(0.70–0.83)	0.70	(0.65–0.80)	0.053
LVEDD (cm)	4.4	(4.1–4.6)	4.2	(3.9–4.4)	0.110
LVPWd (cm)	0.80	(0.70–0.80)	0.70	(0.60–0.70)	0.005*
EF (%)	62.0	(58.1–64.0)	64.3	(62.1–66.1)	0.004*
LVMI (g/m^3^)	29.8	(26.0-35.9)	29.8	(25.7–32.9)	0.526
LV Hypertrophy (mmHg)	6	(27.3%)	8	(19.5%)	0.534
Mean 24-hour SBP (mmHg)	119	(115–123)	108	(105–112)	< 0.001*
Mean 24-hour DBP (mmHg)	74	(71–78)	69	(66–71)	< 0.001*
Mean daytime SBP (mmHg)	124	(120–129)	115	(111–118)	< 0.001*
Mean daytime DBP (mmHg)	81	(77–83)	74	(71–76)	< 0.001*
Mean night-time SBP (mmHg)	110	(106–117)	97	(95–102)	< 0.001*
Mean night-time DBP (mmHg)	66	(63–70)	60	(57–63)	< 0.001*
Non dipper	9	(40.9%)	9	(22.0%)	0.147

Body Mass Index z-score (BMI z-score), Tri-ponderal Mass Index (TMI), Waist Circumference (WC), Waist-to-Height Ratio (WHR), A Body Shape Index (ABSI), Homeostatic Model Assessment Index– Insulin Resistance (HOMA-IR), Homeostatic Model Assessment Index -β (HOMA-β), Quantitative Insulin sensitivity Check Index (QUICKI), Systolic Blood Pressure (SBP), Diastolic Blood Pressure (DBP), Interventricular Septum thickness (IVSd), Left Ventricular Internal Dimensions at end Diastole (LVEDD), Left Ventricular Posterior Wall thickness (LVPWd), Ejection Fraction (EF), Left Ventricular Mass Index (LVMI), Left Ventricular (LV), * *P* < 0.050

Table4 shows a Spearman?s correlation heatmap. WHR was the only central adiposity index that significantly correlated with LVMI.

**Table 4:** Correlation of anthropometric, adiposity, glycometabolic indices, echocardiogram, and ABPM parameters

		Age	BMI z-score	TMI	WC	WHR	ABSI	HOMA-IR	HOMA-β	QUICKI	Office SBP	Office DBP	IVSd	LVEDD	LVPWd	EF	LVMI	Mean 24-hour SBP	Mean 24-hour DBP	
Age	ρ	1,000																		
	P																			ρ
BMI z-score	ρ	−0,390	1,000																	−1,00
	P	0,107																		−0,75
TMI	ρ	−0,054	**0,787**	1,000																−0,50
	P	1,000	< 0,001																	−0,25
WC	ρ	**0,635**	0,147	0,203	1,000															0,00
	P	< 0,001	1,000	1,000																0,25
WHR	ρ	0,072	**0,535**	**0,606**	**0,626**	1,000														0,50
	P	1,000	< 0,001	< 0,001	< 0,001															0,75
ABSI	ρ	−0,065	−0,212	−0,376	0,281	0,397	1,000													1,00
	P	1,000	1,000	0,182	1,000	0,091														
HOMA-IR	ρ	0,302	0,230	0,238	**0,453**	0,192	−0,161	1,000												
	P	1,000	1,000	1,000	0,012	1,000	1,000													
HOMAβ	ρ	0,339	0,132	0,195	**0,516**	0,236	−0,070	**0,835**	1,000											
	P	0,510	1,000	1,000	< 0,001	1,000	1,000	< 0,001												
QUICKI	ρ	−0,285	−0,228	−0,236	**−0,429**	−0,181	0,161	**−0,992**	**−0,819**	1,000										
	P	1,000	1,000	1,000	0,030	1,000	1,000	< 0,001	< 0,001											
Office SBP	ρ	0,407	0,121	0,150	**0,627**	0,311	0,056	**0,415**	**0,425**	**−0,425**	1,000									
	P	0,059	1,000	1,000	< 0,001	1,000	1,000	0,045	0,031	0,030										
Office DBP	ρ	0,285	−0,054	−0,039	0,381	0,039	0,017	0,275	0,208	−0,281	**0,578**	1,000								
	P	1,000	1,000	1,000	0,153	1,000	1,000	1,000	1,000	1,000	< 0,001									
IVSd	ρ	**0,515**	−0,119	−0,012	**0,457**	0,094	−0,055	0,186	0,257	−0,173	0,405	0,258	1,000							
	P	< 0,001	1,000	1,000	0,013	1,000	1,000	1,000	1,000	1,000	0,077	1,000								
LVEDD	ρ	**0,625**	−0,183	−0,120	**0,528**	0,053	0,007	0,155	0,161	−0,125	0,348	0,267	0,307	1,000						
	P	< 0,001	1,000	1,000	< 0,001	1,000	1,000	1,000	1,000	1,000	0,451	1,000	1,000							
LVPWd	ρ	**0,524**	−0,132	−0,120	**0,514**	0,083	0,112	0,406	**0,447**	−0,397	**0,547**	0,308	**0,653**	0,292	1,000					
	P	< 0,001	1,000	1,000	0,001	1,000	1,000	0,074	0,017	0,102	< 0,001	1,000	< 0,001	1,000						
EF	ρ	0,029	−0,254	−0,233	−0,146	−0,215	−0,051	−0,185	−0,055	0,190	−0,250	−0,107	−0,075	0,022	−0,117	1,000				
	P	1,000	1,000	1,000	1,000	1,000	1,000	1,000	1,000	1,000	1,000	1,000	1,000	1,000	1,000					
LVMI	ρ	0,115	0,108	0,213	0,128	0,291	0,024	−0,038	−0,006	0,053	0,196	0,067	**0,494**	0,412	0,380	−0,078	1,000			
	P	1,000	1,000	1,000	1,000	1,000	1,000	1,000	1,000	1,000	1,000	1,000	0,002	0,060	0,171	1,000				
Mean 24-hour SBP	ρ	0,275	0,093	−0,043	0,342	−0,041	−0,141	0,286	0,324	−0,285	**0,466**	0,079	0,327	0,230	**0,454**	−0,138	0,067	1,000		
	P	1,000	1,000	1,000	0,821	1,000	1,000	1,000	1,000	1,000	0,015	1,000	1,000	1,000	0,024	1,000	1,000			
Mean 24-hour DBP	ρ	0,195	−0,121	−0,195	0,103	−0,123	0,064	0,120	0,057	−0,109	0,280	−0,003	0,160	0,101	0,340	−0,173	0,005	**0,583**	1,000	
	P	1,000	1,000	1,000	1,000	1,000	1,000	1,000	1,000	1,000	1,000	1,000	1,000	1,000	0,809	1,000	1,000	< 0,001		

Body Mass Index z-score (BMI z-score), Tri-ponderal Mass Index (TMI), Waist Circumference (WC), Waist-to-Height Ratio (WHR), A Body Shape Index (ABSI), Homeostatic Model Assessment Index– Insulin Resistance (HOMA-IR), Quantitative Insulin sensitivity Check Index (QUICKI), Homeostatic Model Assessment Index -β (HOMA-β), Systolic Blood Pressure (SBP), Diastolic Blood Pressure (DBP), Interventricular Septum thickness (IVSd), Left Ventricular Internal Dimensions at end Diastole (LVEDD), Left Ventricular Posterior Wall thickness (LVPWd), Ejection Fraction (EF), Left Ventricular Mass Index (LVMI), *P* < 0.050 in bold. Multiple testing adjustment was performed according to Bonferroni-Holm

Table5 Shows sex- and age-adjusted association between adiposity indices and left ventricular hypertrophy.

**Table 5:** Waist circumference and WHR were the only central adiposity indices that were significantly associated with left ventricular hypertrophy

	BMI z-score	TMI	WC	WHR	ABSI
Female sex	0.009	0.527	0.006	0.204	0.051
Age (years)	−0.031	0.138	*−0.697	0.250	0.168
BMI z-score	*−1.342				
TMI		*−0.334			
WC			*−0.131		
WHR				*−22.238	
ABSI					−28.186
Constant	5.620	5.969	5.777	*12.010	1.804
P value	0.085	0.073	*0.021	*0.022	0.745
Pseudo R2	0.96	0.101	0.141	0.140	0.018
AIC	70.5	70.2	64.7	67.1	74.1

Associations of adiposity indices with the left ventricular hypertrophy

Body Mass Index z-score (BMI z-score), Tri-ponderal Mass Index (TMI), Waist Circumference (WC), Waist-to-Height Ratio (WHR), A Body Shape Index (ABSI), Akaike information criterion (AIC)

* *P* < 0.050

## Discussion

To the best of our knowledge, this is the first study evaluating the association between TMI and LVH in a group of children and adolescents with obesity. In the studied cohort, patients affected by obesity with LVH had significantly higher BMI z-score, WHR and TMI compared to the group without LVH. A higher TMI is therefore associated with LVH, though TMI, unlike WHR, did not correlated with left ventricular mass index.

It is well known that pediatric onset obesity is related to both target organ damage in the short term, and increased CVD risk and early mortality in adulthood [35, 36]. Therefore, prevention and treatment of obesity and its related detrimental health consequences is essential, starting from pediatric age. Several cardiovascular conditions may affect children with obesity, especially HTN [37] and LVH [8].

Although recent years have seen increased interest in assessing the presence of cardiovascular complications in children and adolescents with obesity, to date there are only few studies evaluating the association of adiposity indices with HTN and LVH.

TMI was originally proposed for its better stability across the age span in children and adolescent, obviating the need for z-scores calculation, and for its improved reliability in identifying body fat compared to BMI [13]. In line with this, Malavazos et al. demonstrated that TMI was superior to BMI as a body fat index [15]. Sun et al., in their systematic review, confirmed that TMI provided similar or better prediction of body adiposity compared to BMI in paediatric age. However, regarding cardio-metabolic outcomes, they found only few studies examining the correlation between TMI and blood pressure. They concluded that either TMI or BMI performed poorly in identifying high BP in children and adolescents, and that their ability varied in different populations [38]. Subsequently, few studies have argued in favour of TMI as a suitable index in discriminating cardio-metabolic risk factors [5, 11]. Alvim et al. showed a strong association between TMI values and CVD risk in a sample of 37.815 Brazilian adolescents (12–17 years old). In fact, adolescents classified as obese (TMI-for-age ≥ 95th percentile) had a higher prevalence of hypertension [11]. In another longitudinal cohort of 36.950 subjects (6–17 years old), the capacity of TMI slightly outperformed BMI in predicting pediatric HTN. The discriminating power of HTN was even stronger when considering the subgroup of subjects aged ≥ 16 years [39].

Previously, we found TMI is a good predictor of high BP among males older than 10 years of age [16], although we assessed exclusively office BP in an outpatient setting. In the present study, focused on a smaller cohort, we didn’t observe an association between TMI and HTN. It is important to note, though, that in the present cohort of subjects HTN was diagnosed using ABPM, obviating the known issue of masked HTN and white coat HTN, particularly common in the pediatric population [9]. We are unaware of previous studies on the association between adiposity indices and HTN diagnosed at ABPM.

HTN is the main cause of LVH in the general population, although it has already been described how in the obese pediatric population LVH can be found independently from HTN [10]. The present study confirms this finding in a population studied extensively with ABPM.

It has long been known in the literature that LVH is associated with obesity, mostly abdominal obesity [40]. As previously reported, TMI is a surrogate index of overall adiposity [13], and it is not a direct measure of visceral or abdominal fat. It is possible that we failed in demonstrating a correlation between TMI and LVMI, since TMI is a surrogate index of total fat and not of fat distribution. On the contrary, in the present work LVMI was significantly associated with WHR and WC. This is not surprising, as WHR and WC are well-known indicators of abdominal fat mass [21]. Recently, WHR showed similar ability in predicting LVH and left ventricular remodeling in a group of hypertensive children and adolescents compared to BMI or WC [23]. Moreover WHR had a better or similar predictive power compared to BMI in identifying eccentric hypertrophy (EH) and concentric hypertrophy (CH) in children aged 6-to-11 years [24], whilst WC performed worst. Hence, WHR has been proposed as a simple and convenient index for screening youth at high risk of target organ damage [23, 24].

As the WHR is an easy measure to perform and to interpret with a fixed cut-off of 0,55 for children, it seems suitable for use in medical practice [21]. Some authors suggested the use of TMI in conjunction with WHR to identify overweight adolescents at high cardio-metabolic risk [15], but further studies need to be conducted.

Finally, it is interesting to note that glycometabolic indices (HOMA- IR, HOMA-β and QUICKI) are strongly associated with the diagnosis of HTN at ABPM in children with obesity [9] but only weakly associated with LVH.

Our study has some limitations: first, a narrow sample size, although homogenous, may have limited the power of statistical tests. Secondly, we have investigated the role of adiposity indices which are surrogate indicators of body fat, whereas a body composition assessment would have been a direct measure of adiposity. It is possible that direct assessment of visceral adiposity distribution (with Dual Energy X-ray Absorptiometry, DEXA) is a better predictor of LVH in children with obesity, but this hypothesis needs to be tested in future studies. Thirdly, inter-observer agreement analysis is not available for auxological evaluation. The ABPM monitoring in every patient, on the other hand, represents the greatest strength of our study.

## Conclusion

TMI, a surrogate indicator of total body fat, is higher in children and adolescents with obesity with LVH when compared to children and adolescents with obesity without LVH, although WC and WHR were significantly associated with LVMI.

### Electronic supplementary material

Below is the link to the electronic supplementary material.

Supplementary Material 1

## Data Availability

Not applicable.
