# Supplementary material for: Tri-ponderal mass index and left ventricular hypertrophy in a cohort of caucasian children and adolescents with obesity
Source: Ital J Pediatr. 2024 Apr 19;50:75. doi: 10.1186/s13052-024-01634-9 (PMC11027303; doi:10.1186/s13052-024-01634-9)
Supplement: Supplementary file 1 — Supplementary Material 1 [file 13052_2024_1634_MOESM1_ESM.docx]

**Reviewer #1:**

*The revised manuscript is acceptable in current form.*

Thank you for your comment.

**Reviewer #2:**

*The authors have responded to all comments.*

*However, I would recommend extending the description of the results.*

*In addition, table 5 lacks description and no reference to adjustment for multiple testing in the tables and results (only stated in method section).*

Thank you for your comment. We have amended the text as per your suggestions.

.
